# Integrated modeling of “soft” and “hard” variables in manufacturing

**DOI:** 10.1007/s00170-022-09872-z

**Published:** 2022-08-09

**Authors:** Mohamed Afy-Shararah, Konstantinos Salonitis

**Affiliations:** Sustainable Manufacturing Systems Centre, Cranfield University, Bedford, Cranfield MK43 0AL England, UK

**Keywords:** Lean manufacturing, Digital modeling, Culture, System dynamics, Discrete event simulation

## Abstract

This paper presents a novel holistic modeling approach for investigating and analyzing the relationship of qualitative variables such as training and absenteeism with quantifiable shopfloor key performance indicators such as quality, inventory, and production rate. Soft variables, supervisor support and work environment, and their relationships with the hard variables, facility layout, and production strategies were investigated in this research. It was found in the literature that increasing absenteeism reduces the rate of production and causes a decrease in motivation, while training can increase the level of motivation if effective. A causal loop diagram was developed based on the evidence in the literature, and a system dynamics simulation model was created to depict these relations. It was confirmed that absenteeism affected the cycle time and motivation inversely, but it was not possible to always maintain a desired level of motivation. A discrete event simulation model was also built for the current and the future state maps of the production system. The model used output from the system dynamics model as its input to investigate the effects of the qualitative variables on the production system performance. This paper discusses in detail the stages of building the simulation models and the results recorded.

## Introduction

The COVID-19 pandemic has added further cost-saving pressures on its production facilities, which leads to even more organizational pressures to be more productive and leaner. Productivity in manufacturing facilities is the result of human behavior and technical decisions because it is a socio-technical system [1]. Human behaviors are typically the result of the organizational culture, employee skills levels, and peer relationships, amongst others. Whereas the technical decisions are typically influenced by the nature of the industry, the products being made, and the requirements of the customers. Using modeling and simulation, this paper investigates the influence that soft variables such as training, absenteeism, motivation, and fatigue, as well as how hard variables such as facility layout and production strategies, have on the manufacturing performance. Modeling and simulation within manufacturing organizations have typically focused on the hard aspects [2], such as scheduling [3], supply chain management [4], and production planning and inventory control [5]. These simulation models often do not consider or factor for the impact of soft aspects associated with human behavior, which makes them less realistic and lacking in their accuracy to reflect the behavior of the real system. On the other hand, the simulation models that focus primarily on the soft aspects tend to be over-simplistic and abstract, offering a reduced level of detail that is essential in the modeling of production systems. As such, and because production systems are identified as socio-technical systems, an accurate model needs to incorporate the soft and hard variables in the same simulation. This paper reviews the soft and hard aspects of production systems and the shop floor employees and offers a solution to combine both types of variables in the same simulation.

## Literature review

Lean manufacturing is an integrated socio-technical system that relies on the human element to accomplish its aim [6], and it aims to add value to the customer by reducing and eliminating the manufacturing waste from the value stream [7]. An effective tool for the identification of manufacturing wastes is value stream mapping (VSM) [8]. Despite its benefits in implementing lean, a VSM assumes a deterministic model and cannot predict the dynamic behavior of a production system. For example, it cannot predict the inventory levels at a given point in time nor the effect of any proposed change on the performance of the system [9]. Simulation has also been identified as a tool to enhance the implementation of lean [10] to address the variation in the system, assess the interaction between system components, and validate alternative future states before implementation to reduce the period of trial-and-error adjustments [9].

Discrete event simulation (DES) is the approach that is more suited for modeling and simulating the technical side of production systems [2], specifically at the shop-floor level. Despite its strengths in representing the dynamics of inventory, throughput, lead times, and process utilization, DES cannot accurately model the social aspect of systems specifically those that relate to human behavior [2]. On the other hand, system dynamics (SD) is the approach that is more suited for modeling and simulating the social side of production systems. Whilst SD has been used in the assessment of manufacturing performance [11], it is more suited to model and simulate the dynamics of human behavior [12], such as motivation and fatigue [13], stress [14], as well as for assessing the effectiveness of training [15]. However, despite its strengths in representing soft intangible variables [16], SD does not accurately represent the dynamics between the machines, parts, and inventory.

### Soft intangible variables

Numerous factors can influence the performance of the employees on the shop floor. These factors can be referred to as “soft” variables, primarily due to them being intangible, and they include motivation [17], job satisfaction [18], leadership styles [19], absenteeism [20], training and learning [21], employee engagement [19], amongst others [22]. Many of these variables are linked, either directly or indirectly. For example, the levels of employee motivation influence the learning effectiveness of the employees in training [21], and it also affects how willing the employees are to show up for work [20]. Therefore, it is important to understand how the performance of a production system can be affected by the motivation of the shop floor employees, particularly via the effectiveness of training and levels of absenteeism.

Training can be defined as the orderly transitioning of behavior and is generally formal [23]. Training occurs through learning, which can be attributed to education, receiving instructions, and planned development [20]. Training is an important aspect for any manufacturing organization, especially in the industries that have stringent standards to operate within. It has the potential to improve the performance of an individual by increasing their abilities [24]. However, the outcome of the training and ultimately its impact on manufacturing performance is influenced by many variables [21], including the quality of the training program and its effectiveness, but also variables related to the employees, such as their years of experience, education level, resistance to change, motivation, and existing skill set.

Absenteeism can be defined as a pattern of being absent from work without any notice or prior information. This can also be termed “unplanned absenteeism.” It can be used to indicate a low degree of commitment and motivation. Employees who are not satisfied with their workplace conditions or recognition for their work can be expected to have a higher level of absenteeism [25]. Absenteeism can be seen to cause a decrease in the production rate because it causes an increase in cycle time, hence a decrease in throughput due to replacement workers [26]. The reduced production rate causes an increase in the planned hours of overtime to fulfill the production loss [27]. This increase in overtime increases the tiredness of the employee, which causes errors, rework, and scrap. Consistent tiredness causes a decrease in employee motivation [28]. Other key variables that influence absenteeism include organizational commitment, supervisor support, job satisfaction, and work environment.

A complex socio-technical system such as a production system cannot be accurately modeled using a single technique, such as DES or SD. The variables related to material flow are more suited to DES, while the variables related to human behavior are more suited to SD. Therefore, a hybrid modeling and simulation approach is more suitable for these problems [2], which will be shown in the following section.

## Model building

**Fig. 1:**
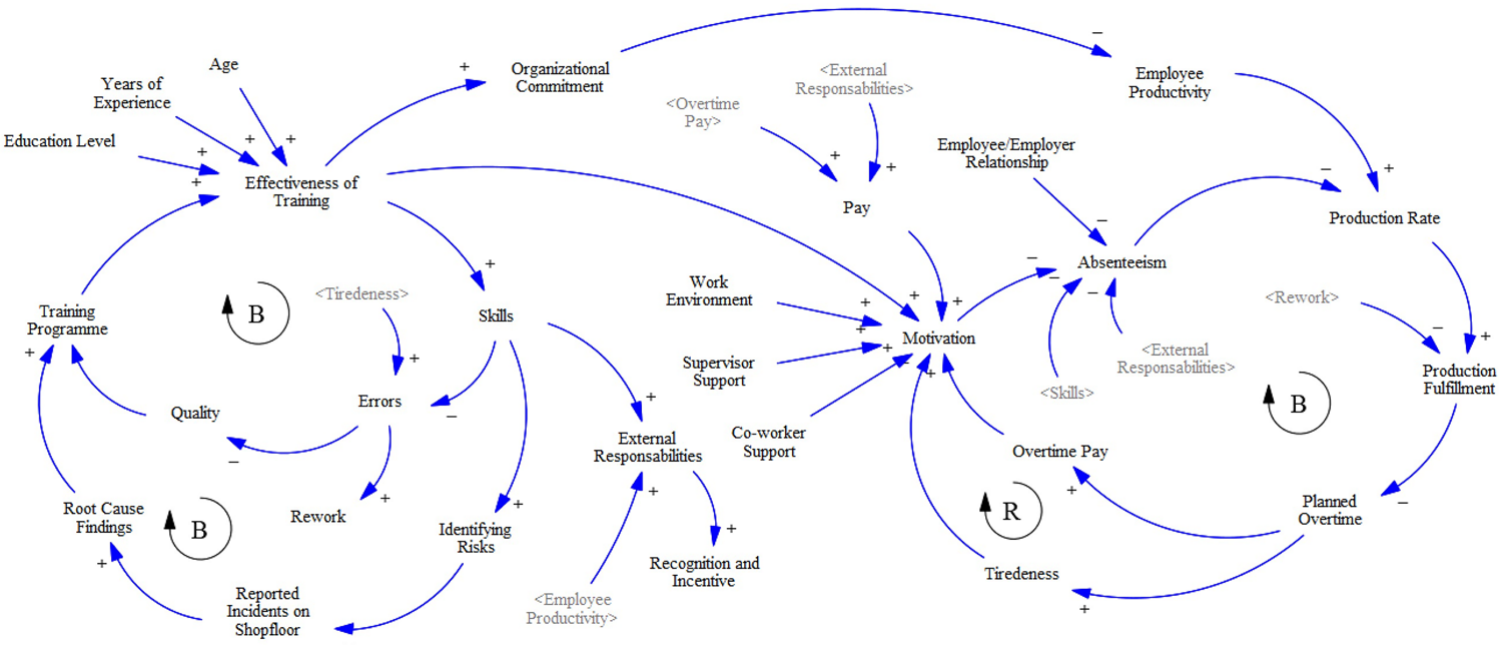
Causal loop diagram (CLD)

**Fig. 2:**
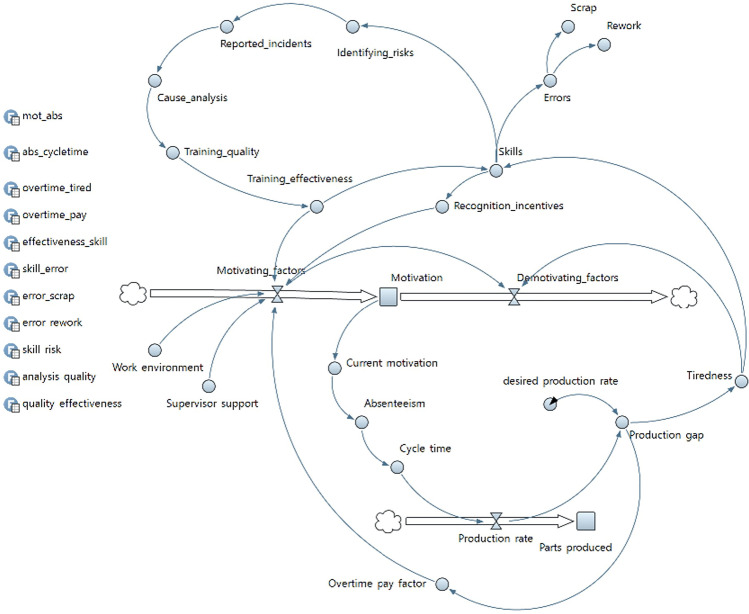
Stock and flow diagram

To understand the impact of the soft intangible variables on manufacturing performance, the first step was to develop and validate a well-defined causal loop diagram (CLD) using the existing literature as well as from expert opinion of stakeholders from the system being studied (Fig. 1). To understand the multiple connections between the various variables identified, a CLD model was initially developed from the literature, which then was refined and developed further over multiple iterations with key stakeholders. However, like the VSM, the CLD is static, and it cannot represent or show the strength of each relationship, which is key for simulating the correct dynamics of the manufacturing system. Therefore, the CLD has been used to develop a stock and flow diagram (SFD) to be solved using SD (see Fig. 2). The strengths of the connections between the variables and their direct effects were assessed using the experts’ opinions, surveys, and the literature when no knowledge was generated from former two sources. The output from the SFD model—in the form of values plotted over time—would then be fed as inputs to a DES model of the production system to be used for running more realistic scenarios that integrate both the soft and the hard variables in the same simulation run.

### System dynamics model

A system dynamics model uses quantifiable variables to allow results to be generated to analyze the system under study. Stocks and flows, which define the accumulation and movement of resources over time, are central to understanding and visualizing the dynamic nature of a complex system [22]. The SFD model was developed and validated with the decision-makers associated with the system under study. The model helps identify and understand the dynamics of employee motivation and to track the changes in their values over time.

Tiredness levels are directly influenced by the gap from the production target, which also affects the overtime and overtime pay. Overtime pay is based on the hours of overtime and the hourly wage. Training effectiveness is based on the quality of the training program [21]. An increase in the training effectiveness increases the skill, which allows for better recognition of the employee and results in increasing motivation over time [20]. The current level of motivation has an impact on the commitment levels of the employees, and hence their absenteeism [19]. Absenteeism affects the cycle time since there is either a replacement worker or the absence of a worker at the station, which causes an increase in the cycle time that causes a change in the production rate.

Values for the work environment, supervisor support, and recognition incentives were measured on a scale of 1–5. These values were obtained based on a survey administered specifically to measure employee engagement, and grouped based on work environment, supervisor support, job satisfaction, and recognition and responsibilities. The values were calculated by taking the average of the scores of the questions within each group. Engagement here can be defined as the relation between the employee, the organization, and the people at a higher working post. The survey has a set of 12 questions to assess an employee’s level of engagement and understand their level of motivation. Some of the key parameters that have been considered to develop the questionnaire for describing engagement and motivation are the availability of a conducive work environment, the availability of opportunities for external responsibilities, the level of participation encouraged by the organization, the provision for training of the employees for skill development, and the benefits and bonuses provided.

### Discrete event simulation model

**Fig. 3:**
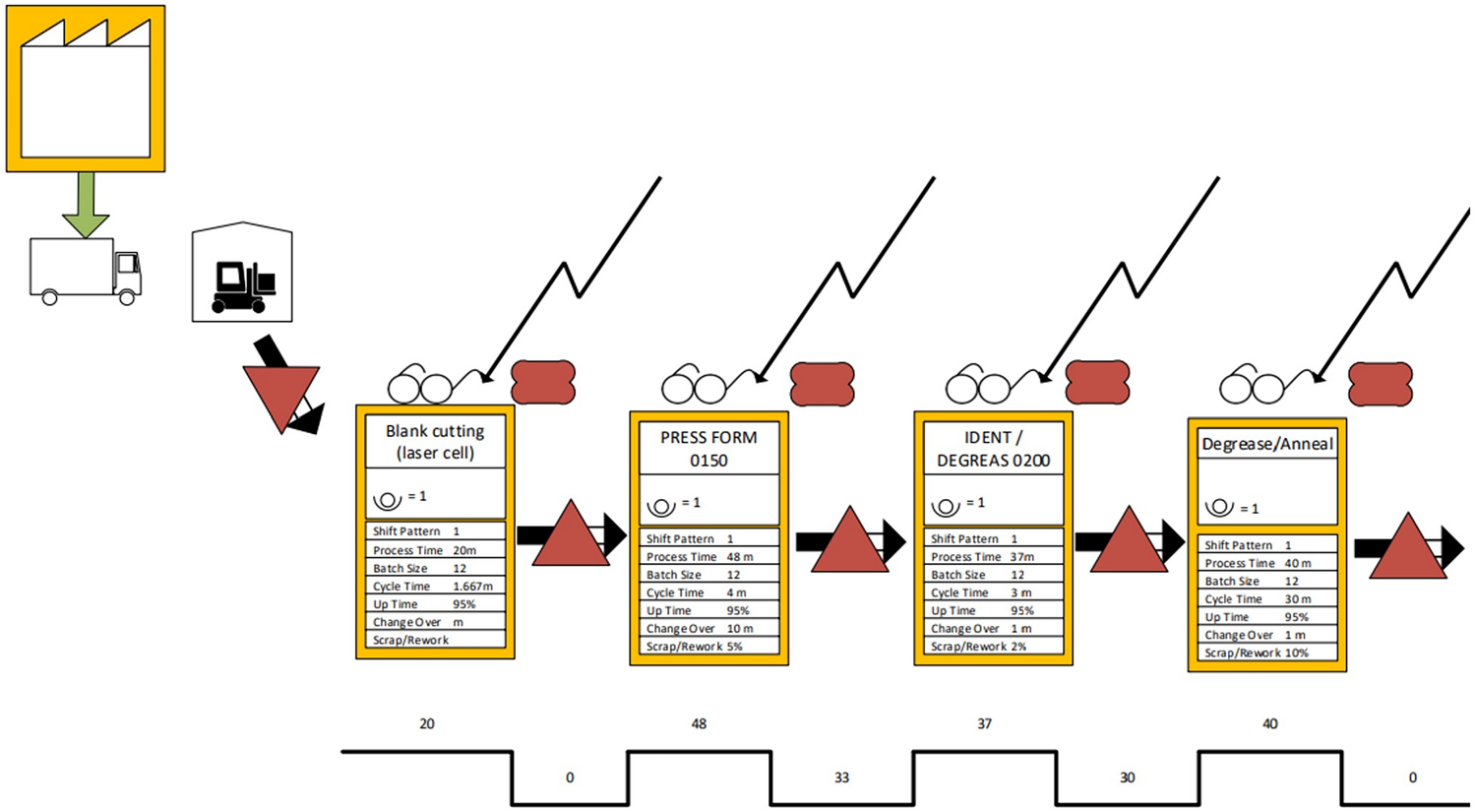
A snapshot of the value stream map

**Fig. 4:**
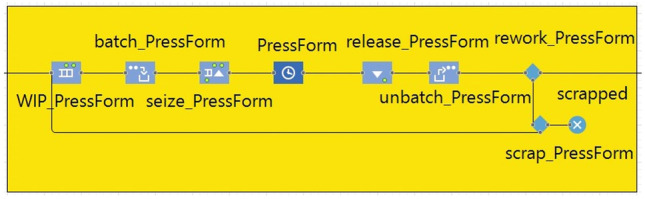
Section of discrete-event model

The DES model was developed using AnyLogic simulation software and was built to replicate the existing VSMs developed according to lean principles to understand the flow of materials and identify areas of potential lean manufacturing waste, and suggested future state maps. The production system being modelled is one in the aerospace industry. The component produced is a titanium duct that currently goes through 58 linear processes. This includes outsourcing a few processes to subcontractors. The part goes through one process after another in a succession, and there is inventory storage after each of the processes. The production processes are located in various buildings at the production facility. The production facility operates an 8-h shift, and supplier deliveries arrive on the first day of each week. For clarity, consistency, and presentation purposes, the VSMs were digitalized, which provided the groundwork to create the DES simulation model. A snapshot of the VSM is shown below (see Fig. 3), and an example of the modeling blocks and modeling logic used to replicate the VSM with a discrete event simulation model (see Fig. 4). All the processes were replicated using the same logic. “Queues” were used as WIP storage, which is then batched according to the process’ “batch” size, and after spending the processing time represented by a “delay,” it will be “unbatched,” and either reworked, scrapped, or cleared to proceed to next process. To process any batch, an employee needs to be available, which is represented by a “resource.” Once the process is finished, the employee/resource is released until needed again. The manufacturing information used included process time, cycle time, batch size, up time for the resources (machines), change over time, scrap rate, working hours, number of shifts in a day, information regarding the transportation of parts, as well as the arrival rate from suppliers. Assumptions were made regarding the weekly arrivals of parts, the triangular distribution of the processing time with the min and max calculated as 15% deviation, the percentage of rework as 25% of scrap, and the speed of transportation as 1 m/s.

The model was run for a period of 1 year (excluding warm-up period) and was replicated for 50 runs. The validation and verification are both required in any simulation project. When the model behaves in the way it should and as intended by the modeler, then it can be considered verified, which was the case. If the model is an accurate representation of the actual system, then it is considered valid. The model behavior after running was compared to the behavior of the real system, and they were considered matching, in terms of values and by the opinions of experts of the real system.

The criteria employed to compare the performance of the system under different scenarios were cash, quality, and delivery. The amount of work in process (WIP) in the system would reflect the amount of cash tied up on the shop floor. The WIP/cash was calculated as the average quantity of parts present in the model throughout the simulation run. Quality was calculated as the sum of the number of defective parts produced at each process about the total number of parts over the whole simulation run. Delivery is the rate of production associated with each scenario and has been calculated as the time in minutes between each part exiting the system.

## Results and analysis

This section discusses the results of different scenarios purposively developed to reflect the different states of the system. Having the ability to exchange model variables between the DES and SD models has allowed for the inclusion of the social behavior on the material flow within the production system, which otherwise would not have been possible using either of the simulation techniques on its own. The scenarios included the as-is current state map, proposals related to future state improvements, as well as the impact of the soft qualitative variables, work environment, and supervisor support, on the production performance. The five scenarios and the criteria differentiating between them can be seen in Table 1. The soft variables can have values ranging from 0 to 5, with 0 being the lowest and 5 being the highest, and all the scenarios were analyzed regarding the average utilization of the processes (Table 2), the total amount of WIP in the system, which is leading to increased lead times, the quality levels, and the rate of production (or delivery).

**Table 1:** The criteria differentiation the simulation scenarios

Criteria	Scenario 1	Scenario 2	Scenario 3	Scenario 4	Scenario 5
Current state map	Yes	Yes	Yes	Yes	X
Transport batch size	X	50%	X	X	X
Distance traveled	X	50%	X	X	X
Work environment	X	X	High	High	X
Supervisor support	X	X	High	Low	X
Future-state map	X	X	X	X	Yes

**Table 2:** Simulated results for each of the scenarios

KPI	Scenario 1	Scenario 2	Scenario 3	Scenario 4	Scenario 5
Avg. process utilization	34.9%	35.3%	42.7%	26.5%	80.5%
Cash (WIP)	1150	1176	1300	1520	387
Quality (Yield %)	75.1%	74.9%	71.6%	60.2%	92.8%
Delivery (mins btw. parts)	1117	1055	1273	1713	2112

The future state map suggested aims to improve the efficiency of the production line by switching to a different production system by reducing the number of processes through merging, which currently is a linear production line and contributes significantly to a long time from receiving the raw material to shipment. Unlike the push system used in the current state model, the future model aims to use a pull and Kanban system but still uses a linear production line. The pull system allows to focus production on customer demand and produce what is needed only to reduce the number of parts in inventory as well as try to optimize the process utilization. It also includes the implementation of first-in-first-out (FIFO) lanes that have a limited capacity to prevent overproducing.

### Scenario 1

Shows poor utilization of the overall system, which is attributed to the significant variation in processing times and having no controlled or managed WIP to ensure smooth flow of materials. However, there is a high level of WIP reported in the system, albeit not strategically located to manage the flow; instead they are located before the bottlenecks. The quality level reported shows an average level of quality output, and this is attributed to the facility layout and the poor levels of quality of some processes. Finally, the rate of production can be attributed to the amount of inventory in the system as well as the processing times of some of the processes.

### Scenario 2

A variation in the process’ utilization prompted an investigation to identify whether this was because of transportation waste resulting from a poor facility layout. As such, the scenario tested the hypothesis that transportation waste contributed significantly to the current levels of performance. Therefore, using the as-is current state but reducing the distances between the processes and the transportation batch sizes by 50%, there was found to be no improvement in the performance metrics of interest. Hence, changing the layout would not be the best scenario to be adopted.

### Scenarios 3 and 4

Scenario 1 could be considered an “idealistic” version of the current state map because it does not factor for any of the “realistic” human behaviors that govern any socio-technical system, of which manufacturing facilities are one of them. Both scenarios investigate the impact of qualitative variables on the performance of the operator and hence on the production system. The inclusion of these factors makes the model better suited for predicting outcomes on the actual shop floor, i.e., the deviation from the idealistic production values occurs due to the unavoidable effect of tiredness in employees over time. It was found that when the level of support from supervisors is high, the production system performed closer to its idealistic values. In this case, absenteeism was less and the effect of tiredness is not that alarmingly high. In terms of manufacturing performance, the average process utilization is lower in scenario 4 due mostly to the high operator absence levels caused by poor support and work environment, which is the opposite case in scenario 3 due to the higher levels of motivation the employees have. This results in a decrease in the completion of jobs and a difference in production rates, which when the low will also show in the amount of WIP on the shop floor. The tiredness that the operators experience and the impact it has on their production quality is also evidenced in the quality level.

### Scenario 5

This scenario replicates the proposed future state map. The main improvement in performance was the significant reduction in WIP levels and the increase in utilization due to the merging of some processes and inventory control techniques. The yield levels are recorded to be higher due to the presence of fewer processes in a product layout, which reduces the overall number of defects. The regular presence of a controlled amount of WIP that is consumed whenever there is demand ensures that the processes are not idle, and hence the utilization levels are higher. The presence of a supermarket inventory coupled with a PULL system results in lower inventory levels. Finally, there is a clear increase in the number of minutes per part in comparison with all the other scenarios. This can be seen as counterintuitive, especially after the implementation of both FLOW and PULL systems. Nevertheless, this is due to the imbalance created within the production line due to the inappropriate merging of some of the processes, which led to an increase in processing time and the creation of new bottlenecks.

## Conclusion

Literature review on management psychology allowed the identification of various causes and effects of variables like training, absenteeism, and motivation and the possible impact that they would have on manufacturing performance. The simulation confirmed how the various production KPIs were affected by the soft qualitative variables and showed the extent of their influence. It could be concluded that tiredness seemed to have an overpowering effect on the whole system, causing a decline in the level of motivation of the employees. After testing the impact of the soft variables, supervisor support, and working environment on the current state of the system, it has been shown that the level of supervisor support to the operator and their social wellbeing is more effective in production terms than in the workplace organization and technical 5S, etc. We recommend providing high levels of both to increase the motivation, quality yield, and production rates and that it would be wiser for companies to invest similarly in efforts to improve the work environment and increase supervisor support. The simulation analysis resulted in the identification of lean waste, such as overproduction, overprocessing, transportation, and inventory. It was shown that with the current production system configuration, the transportation waste did not have a significant impact. The presence of a linear production line, with significant differences in the production speeds, has shown to have a major impact on the utilization and the WIP, therefore, careful consideration of processes merging, and line balancing is strongly recommended. Further studies interested in continuing research in this domain would be encouraged to investigate the rate of skills development, the impact of new starters, as well as other KPIs such as safety and the cost of production.

## Data Availability

Data transparency the data used in this paper can be obtained by contacting the corresponding author.

## Code availability

The code used in this paper can be obtained by contacting the corresponding author.
